# Tunable Magnetocaloric Properties of Gd-Based Alloys by Adding Tb and Doping Fe Elements

**DOI:** 10.3390/ma12182877

**Published:** 2019-09-06

**Authors:** Lingfeng Xu, Chengyuan Qian, Yongchang Ai, Tong Su, Xueling Hou

**Affiliations:** 1 Laboratory for Microstructures of Shanghai University, Shanghai 200444, China; 2 School of Materials Science and Engineering, Shanghai University, Shanghai 200072, China

**Keywords:** Gd_1−*x*_Tb*_x_*, (Gd_0.73_Tb_0.27_)_1−*y*_Fe*_y_*, magnetocaloric effect, low magnetic field, adjustable Curie temperature

## Abstract

In this paper, the magnetocaloric properties of Gd_1*−x*_Tb*_x_* alloys were studied and the optimum composition was determined to be Gd_0.73_Tb_0.27_. On the basis of Gd_0.73_Tb_0.27_, the influence of different Fe-doping content was discussed and the effect of heat treatment was also investigated. The adiabatic temperature change (ΔT*_ad_*) obtained by the direct measurement method (under a low magnetic field of 1.2 T) and specific heat capacity calculation method (indirect measurement) was used to characterize the magnetocaloric properties of Gd_1−*x*_Tb*_x_*(*x* = 0~0.4) and (Gd_0.73_Tb_0.27_)_1−*y*_Fe*_y_* (*y* = 0~0.15), and the isothermal magnetic entropy (ΔS*_M_*) was also used as a reference parameter for evaluating the magnetocaloric properties of samples together with ΔT*_ad_*. In Gd_1*−x*_Tb*_x_* alloys, the Curie temperature (T_c_) decreased from 293 K (*x* = 0) to 257 K (*x* = 0.4) with increasing Tb content, and the Gd_0.73_Tb_0.27_ alloy obtained the best adiabatic temperature change, which was ~3.5 K in a magnetic field up to 1.2 T (T_c_ = 276 K). When the doping content of Fe increased from *y* = 0 to *y* = 0.15, the T_c_ of (Gd_0.73_Tb_0.27_)_1−*y*_Fe*_y_* (*y* = 0~0.15) alloys increased significantly from 276 K (*y* = 0) to 281 K (*y* = 0.15), and a good magnetocaloric effect was maintained. The annealing of alloys (Gd_0.73_Tb_0.27_)_1−*y*_Fe*_y_* (*y* = 0~0.15) at 1073 K for 10 h resulted in an average increase of 0.3 K in the maximum adiabatic temperature change and a slight increase in T_c_. This study is of great significance for the study of magnetic refrigeration materials with adjustable Curie temperature in a low magnetic field.

## 1. Introduction

Magnetic refrigeration technology based on magnetocaloric effect (MCE) has gained wide attention because of its high efficiency and low carbon dioxide emissions. As a new refrigeration technology, its environmentally friendly properties can largely reduce the global greenhouse effect and excessive energy consumption. The MCE is the endothermic and exothermic behaviors of materials with a change in the applied magnetic field, and it is evaluated by adiabatic temperature change (ΔT*_ad_*) and isothermal magnetic entropy (ΔS*_M_*). The ideal operating temperature of the magnetic refrigeration material is near the Curie temperature, because the adiabatic temperature change and the isothermal magnetic entropy reach peaks in this temperature range.

Room-temperature magnetic refrigeration technology is expected to replace traditional gas compression refrigeration technology in the future, therefore, magnetic materials with excellent magnetocaloric properties at room temperature have been extensively studied around the world [1,2,3,4]. After decades of research, many excellent room temperature magnetic refrigeration materials have been discovered. In 1968, Brown [5] found the large MCE of Gd (T_c_ = 293 K). In 1997, Pecharsky and Gschneidner [6] observed Gd_5_Si_2_Ge_2_ with a first-order phase transition having an MCE of about 18 J/(kg·K) under the magnetic field change of 0–5 T, which is larger than that of pure Gd (~10 J/(kg·K)) under the same condition. Fengxia Hu et al. [7] found that LaFe_13−*x*_Si*_x_* had a large MCE of about 19.7 J/(kg·K) at 208 K for a field change of 0–5 T. In addition to the discoveries mentioned above, materials such as MnAs_1−*x*_Sb*_x_* [8,9], Ni–Mn–Sn [10,11,12], and La_1−*x*_Ca*_x_*MnO_3_ [13,14,15] have also been found to have good room temperature magnetic refrigeration performances. These achievements are sufficient to show that room temperature magnetic refrigeration has good development prospects, especially the pure Gd, which is now used as a benchmark for magnetic refrigeration materials. 

However, the isothermal magnetic entropy (ΔS*_M_*) obtained by calculating the isothermal magnetization curve using Maxwell’s equation (Equation (1)) is limited. The results obtained by indirect measurement show that the magnetic entropy change calculated by Maxwell equation should not have a huge peak value. Further research shows that the calculation of magnetic entropy change by Maxwell’s equation is not applicable near Curie temperature, because paramagnetism (PM) and ferromagnetism (FM) coexist near Curie temperature, so that the huge false results of entropy peak are obtained [16]. Therefore, it is not appropriate to use isothermal magnetic entropy to evaluate the magnetocaloric properties of a material. The results of ΔS*_M_* can be used as a reference for magnetocaloric properties. In order to correctly evaluate the magnetocaloric properties of magnetic materials, it is necessary to study the performance of the material under cyclic conditions [17]. 

The adiabatic temperature change (ΔT*_ad_*) measured by direct measurement method (i.e., ΔT*_ad_* is the difference among the temperature of the sample measured directly at H_i_ and H_f_, where H_f_ and H_i_ are the final and initial magnetic fields, respectively) and specific heat capacity calculation method (indirect measurement) is also suitable for practical applications [18]. Adiabatic temperature change is the driving force of the heat transfer efficiency of the heat transfer fluid in the refrigerator. The adiabatic temperature change is also a key and direct parameter to measure the magnetocaloric properties of a material. It is more direct and accurate to characterize the magnetocaloric properties of materials through adiabatic temperature change. 

The direct measurement method is more suitable for the testing of commercial products because of its intuitiveness and convenience. The indirect measurement method is applicable to the equilibrium state or the near equilibrium state. However, most of the magnetic refrigeration processes are dynamic, so the ΔT*_ad_* obtained by the indirect measurement method was used as supplementary data and reference for the direct measurement method in this paper [2]. These two methods can be applied in both first-order and second-order phase transition magnetic materials. 

The premise of the excellent MCE in the currently available magnetic refrigerant materials is only realized in a high-cost superconducting magnetic fields (usually from 0 T to 5 T/10 T), which brings out high costs in practical application. Therefore, it is very important to develop advanced magnetic refrigeration materials with high adiabatic temperature change under low-applied magnetic fields provided by permanent magnets [19,20]. The adiabatic temperature changes directly measured in this paper were achieved with a 1.2 T low magnetic field provided by an NdFeB permanent magnet.

As a typical magnetic refrigeration material, pure Gd has an excellent application prospect in the field of magnetic refrigeration. However, for the reason that the Curie temperature of pure Gd is fixed and not adjustable, its application scope is limited. Therefore, it is of great significance to study alloys with variable Curie temperature [21,22,23,24]. In this work, Gd and Tb alloys with different atomic ratios were studied, and the effect of adding Tb on the T_c_ and MCE of Gd-based alloys was achieved. After the Gd_1−*x*_Tb*_x_* alloy system was determined, the effects of doping a small amount of Fe and adding heat treatment on the MCE were also revealed.

## 2. Experimental Details

**Figure 1:**
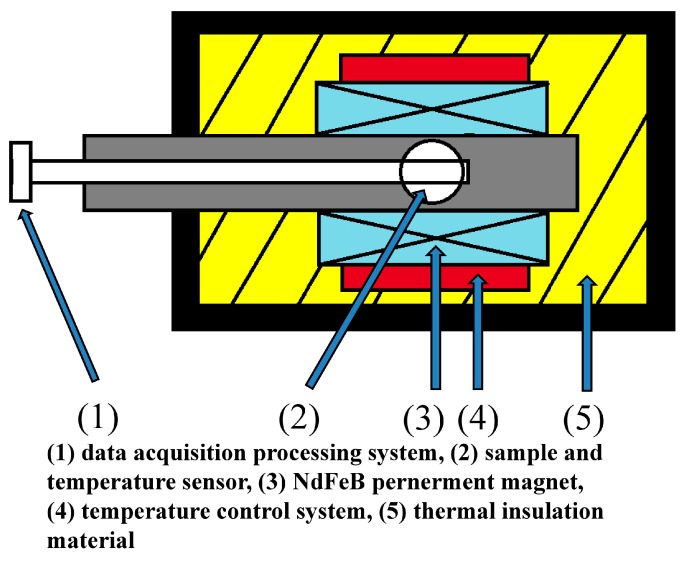
The structure chart of ΔT*_ad_*–T direct measuring instrument.

The alloys of Gd_1−*x*_Tb*_x_*, (Gd_0.73_Tb_0.27_)_1−*y*_Fe*_y_* (*x* = 0~0.4, *y* = 0~0.15, at.%) were obtained by arc melting Gd (99.9%), Tb (99.9%), and Fe (99.9%) in an argon atmosphere. Each ingot was smelted five times to ensure uniformity of composition. Heat treatments of (Gd_0.73_Tb_0.27_)_1−*y*_Fe*_y_* (*y* = 0~0.15) were carried out at 1073 K for 10 h. The phase structure was characterized on a D/max-rB X-ray diffractometer. The adiabatic temperature change (ΔT*_ad_*) of all samples was measured by the direct measurement method under an applied magnetic field of 1.2 T (the magnetocaloric direct measuring instrument is shown in Figure 1. Test procedure: (1) Firstly, the sample was attached to the temperature sensor in an adiabatic thermostat, and then the initial temperature, the end temperature, and heating rate of the test were set; (2) in the second step, the sample was pushed into an applied magnetic field of 1.2 T, and the temperature controller was operated to raise the temperature; (3) in the third step, the temperature of the test chamber rose slowly, and the test was performed every 4 K during the heating process; the instrument pulled the sample out of the magnetic field, the temperature of the sample dropped sharply until it was stable, and the T and ΔT*_ad_* at this time were recorded; (4) the fourth step was to push the sample into the magnetic field until the next test temperature point). 

The physical property measurement system (PPMS-9) was used to measure the samples’ isothermal magnetization curve in a 2 T magnetic field (temperature increment was 4 K). The magnetic entropy change (ΔS*_M_*) calculated by the Maxwell relation (1):

(1) $$\Delta S_{M}={\int_{0}^{H}\left(\partial M/\partial T\right)}_{H}\mathrm{dH}$$

The PPMS system was also used to measure the specific heat capacity in the temperature range of 2–400 K under a zero applied magnetic field. The adiabatic temperature change can also be calculated by applying Formula (2) shown below.

(2) $$\Delta T_{\mathrm{ad}}=\frac{T}{C_{p}}\Delta S_{M}$$

(The parameters in Formula (2)—ΔT*_ad_*: adiabatic temperature change; T: temperature; C_p_: specific heat capacity; ΔS*_M_*: magnetic entropy change.)

## 3. Results and Discussion

### 3.1. Gd–Tb Alloys

**Figure 2:**
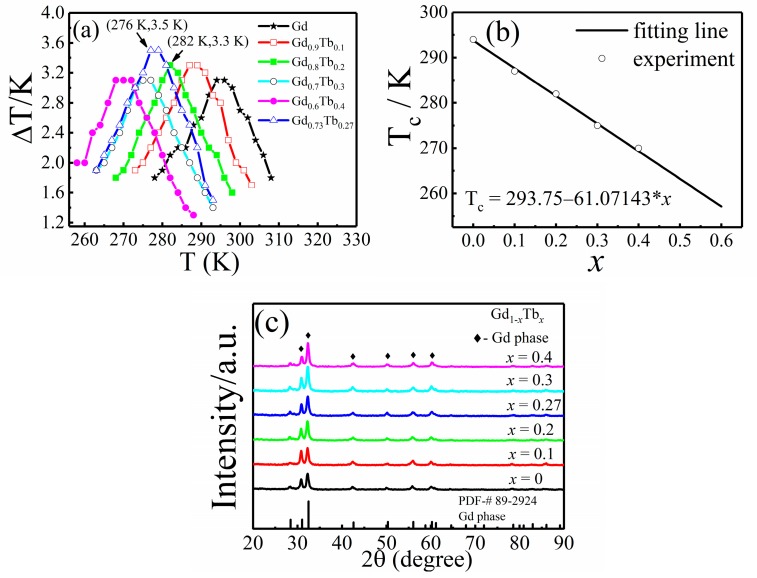
(**a**) Adiabatic temperature change of Gd_1−*x*_Tb*_x_* under a 1.2 T applied magnetic field by direct measurement method, (**b**) Linear fit of the Curie temperature (T_c_) and Tb content (*x*) of Gd_1−*x*_Tb*_x_*, (**c**) X-ray diffraction patterns of the Gd_1−*x*_Tb*_x_* (*x* = 0, 0.1, 0.2, 0.27, 0.3, 0.4) samples.

The adiabatic temperature change (ΔT*_ad_*) (achieved by direct measurement) and the Curie temperature (T_c_) of Gd_1−*x*_Tb*_x_* (the value of *x* from 0 to 0.4, step size is 0.1) are shown in Figure 2a. As the Tb content increased, the Curie temperature decreased monotonously, in accordance with the linear fitting equation shown in Figure 2b. To consider the Curie temperature and the adiabatic temperature change together as a whole, further study is needed between *x* = 0.1 and *x* = 0.3 (the value of *x* from 0.1 to 0.3, step size is 0.01). It can be concluded from Figure 2a that when *x* = 0.27, the Gd–Tb system obtained the largest adiabatic temperature change under 1.2 T applied magnetic field (ΔT*_ad_*= 3.5 K, T_c_ = 276 K). The X-ray diffraction results for Gd_1−*x*_Tb*_x_* alloys (*x* = 0, 0.1, 0.2, 0.27, 0.3, 0.4) are shown in Figure 2c. The results showed that these samples had similar XRD curves, and only Gd phase can be labeled, indicating that the Tb atoms solubilize in Gd solutions. The crystal structure of Gd and Tb was a hexagonal, close-packed structure, and the atomic radius difference of the elements (Δr) was 1.2% (r_Gd_ = 2.54 Å, r_Tb_ = 2.51 Å), which tended to form a substitutional solid solution. 

**Figure 3:**
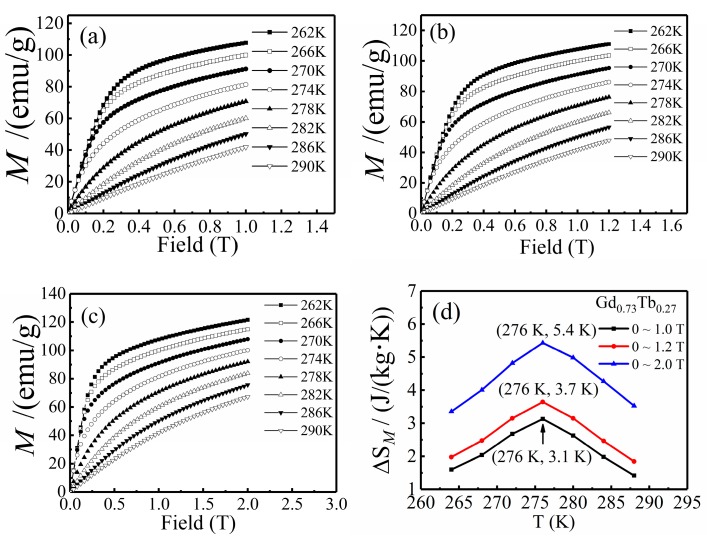
(**a**–**c**) The magnetization isotherms of Gd_0.73_Tb_0.27_ in magnetic fields of 1 T, 1.2 T, 2 T, (**d**) magnetic entropy change of Gd_0.73_Tb_0.27_ from M(*μ*_0_*H*)_T_ dependence using Equation (1) in magnetic fields of 1 T, 1.2 T, and 2 T.

Isothermal magnetization curves M(*μ*_0_*H*)_T_ of Gd_0.73_Tb_0.27_ alloys under different magnetic fields (0–1 T, 0–1.2 T, 0–2 T) were also measured, showing in Figure 3a–c. The isothermal magnetic entropy change (ΔS*_M_*) calculated by Maxwell’s equation (Equation (1)) on the isothermal magnetization curves M(*μ*_0_*H*)_T_ can be used together with the adiabatic temperature change to evaluate the magnetocaloric properties of alloys, which provides a more accurate result. Figure 3d indicates that with the magnetic field increases, the isothermal magnetic entropy increased significantly, reaching the maximum value near Curie temperature and the value were 3.1 J·kg^−1^·K^−1^, 3.7 J·kg^−1^·K^−1^, 5.4 J·kg^−1^·K^−1^, corresponding to the applied magnetic field changes of 0–1 T, 0–1.2 T, 0–2 T. 

**Figure 4:**
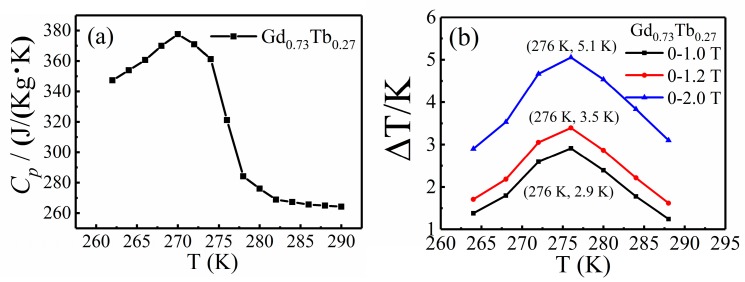
(**a**) Specific heats of Gd_0.73_Tb_0.27_ in zero external field, (**b**) adiabatic temperature change of Gd_0.73_Tb_0.27_ (indirect measurement) in magnetic fields of 1 T, 1.2 T, and 2 T.

The adiabatic temperature change obtained by indirect measurement is also an important parameter for measuring the magnetocaloric properties of materials. In this paper, we used it as a supplement and reference for the results of direct measurement. The parameters used in Equation (2) are isothermal magnetic entropy change (ΔS*_M_*) and specific heat capacity under zero field (C*_p_*). Figure 4a shows the specific heat capacity obtained in zero applied field, Figure 3d shows the isothermal magnetic entropy change, and the result of adiabatic temperature change calculated by combining the data of these two figures is shown in Figure 4b. By comparing the value of the adiabatic temperature change achieved by indirect measurement method and direct measurement method under 1.2 T magnetic field, we found that the peak value was the same, about 3.5 K, which shows that the direct measurement method and the indirect measurement method were in good agreement. In addition, when the magnetic fields were 1 T and 2 T, the values of the adiabatic temperature change obtained by indirect measurement were 2.9 K and 5.1 K, respectively.

### 3.2. Gd–Tb–Fe Alloys 

**Figure 5:**
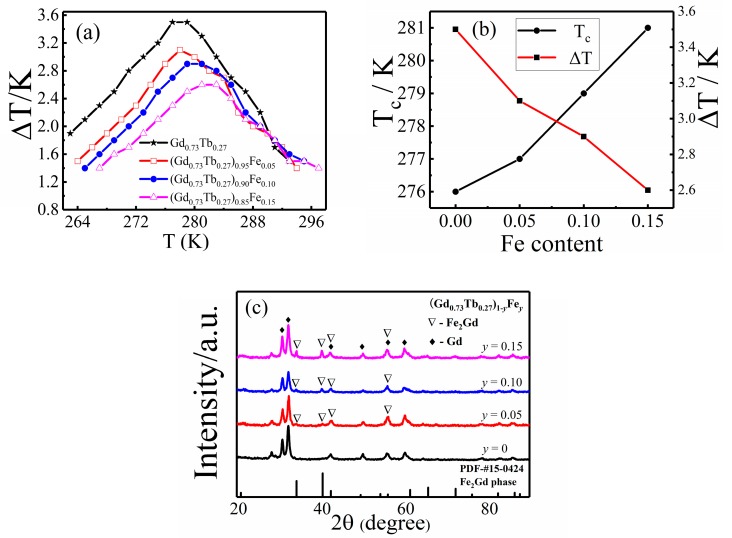
(**a**) Adiabatic temperature change of Gd_1−*x*_Tb*_x_*samples (direct measurement) in a magnetic field of 1.2 T, (**b**) adiabatic temperature change and T_c_ of (Gd_0.73_Tb_0.27_)_1−*y*_Fe*_y_* alloys, (**c**) the XRD patterns of (Gd_0.73_Tb_0.27_)_1−*y*_Fe*_y_* alloys.

According to the research on Gd_1−*x*_Tb*_x_*, Gd_0.73_Tb_0.27_ has the best magnetocaloric effect. On the basis of Gd_0.73_Tb_0.27_, the influence of Fe doping needs to be further studied. The curves of maximal adiabatic temperature change and the Curie temperature varied with the content of Fe (*y* value) can be clearly seen in Figure 5a,b. With the increase of Fe content, the maximum adiabatic temperature change of (Gd_0.73_Tb_0.27_)_1−*y*_Fe*_y_* decreases from 3.5 K (*y* = 0) to 2.6 K (*y* = 0.15) under 1.2 T magnetic field, and the Curie temperature of (Gd_0.73_Tb_0.27_)_1−*y*_Fe*_y_* increases from 276 K (*y* = 0) to 281 K (*y* = 0.15).

The XRD pattern of (Gd_0.73_Tb_0.27_)_1−*y*_Fe*_y_* shown in Figure 5c can reveal the effect of Fe doping on the phase structure. When Fe content *x* = 0.05, Fe_2_Gd phase begins to appear. With the increase of Fe doping, the cubic Fe_2_Gd phase content gradually increases. Because the Curie temperature of Fe_2_Gd phase is too high (T_c_ = 795 K), the Fe_2_Gd phase is still ferromagnetic when the ferromagnetic and paramagnetic transition take place in the main phase at 200–300 K, which weakens the intensity of the magnetic moment change, thus leading to the reduction of the magnetocaloric effect. The magnetic moment of Fe was 5.8, and the spin magnetic moment of Fe was anti-parallel with the spin magnetic moments of Gd and Tb, so that the saturation magnetization was reduced, also resulting in a decrease in adiabatic temperature change.

The transition element Fe had a high Curie temperature due to the strong interaction among 3d electrons, and its Curie temperature was 1043 K. The addition of Fe enhanced the indirect interaction of 4f–4f electrons between Gd and Tb atoms, and the interaction of Fe–Fe was stronger than R–Fe and R–R (R = Gd, Tb). As a result, the Curie temperature increased due to the increase in Fe content. Although the addition of Fe reduced the maximum adiabatic temperature change of the alloy, its ΔT*_ad_* was not lower than pure Gd (ΔT*_ad_* = 3.1 K) under the same applied magnetic field change of 0–1.2 T, the alloy is still an excellent room temperature magnetic refrigeration material, and the addition of Fe can adjust the Curie temperature while reducing costs.

The effect of Fe doping on the magnetocaloric properties was obtained by studying the (Gd_0.73_Tb_0.27_)_1−*y*_Fe*_y_* alloy. The heat treatment of the (Gd_0.73_Tb_0.27_)_1−*y*_Fe*_y_* was also very meaningful and is worth further investigation. According to the binary phase diagram of Gd–Fe and Tb–Fe, the solidus temperature of the two was 1118 K and 1120 K, respectively, thereby determining the heat treatment temperature of 1073 K and the heat treatment time of 10 h, in order to homogenize the structure, remove the residual stress, and reduce the lattice defects.

**Figure 6:**
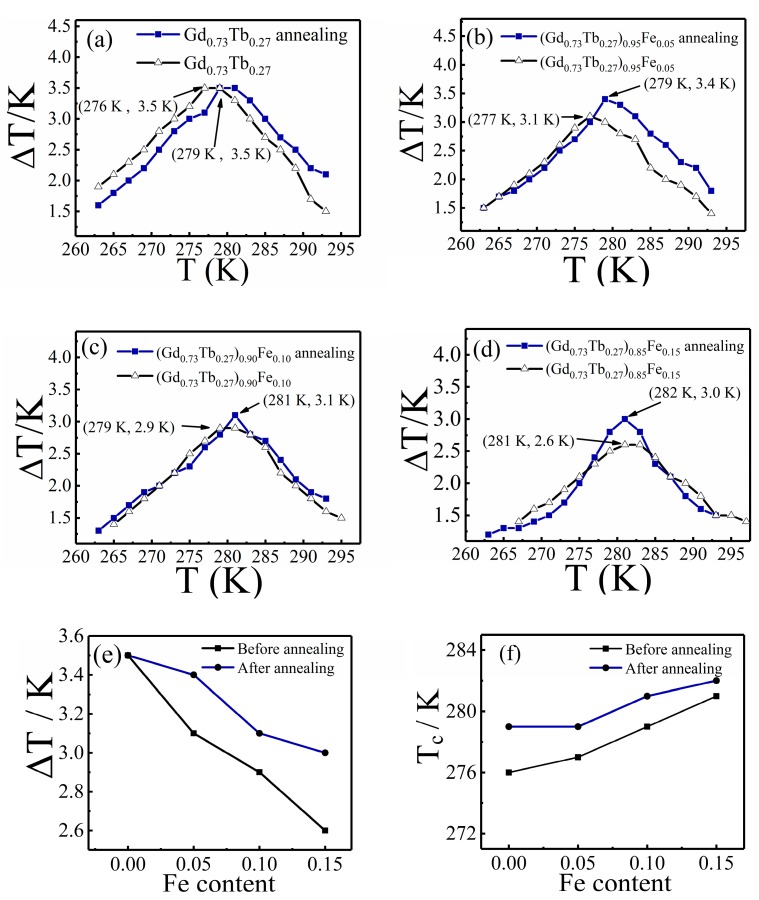
(**a**–**e**) Comparison of the adiabatic temperature change of alloy (Gd_0.73_Tb_0.27_)_1−*y*_Fe*_y_* (direct measurement) before and after heat treatment, (**f**) comparison of the Curie temperature of alloy (Gd_0.73_Tb_0.27_)_1−*y*_Fe*_y_* before and after heat treatment.

As one can see in Figure 6a–d, compared with the adiabatic temperature change curves before and after heat treatment, the maximum adiabatic temperature change of Gd_0.73_Tb_0.27_ did not change, while the others obviously increased. The increase in the maximum adiabatic temperature change can be observed more intuitively from Figure 6e, with an average raise of 0.3 K. The change in T_c_ can be derived from Figure 6f. From the temperature measurement point of view, the Curie temperature rise rarely changed.

**Figure 7:**
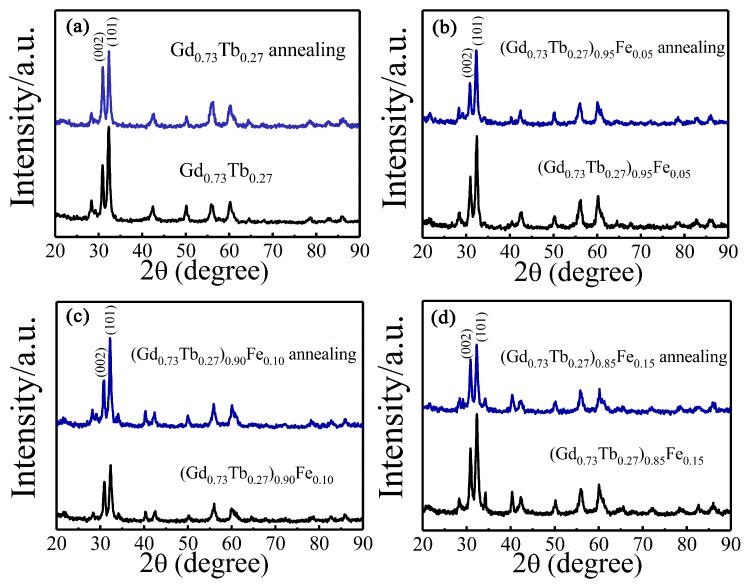
The XRD patterns of comparison of alloy (Gd_0.73_Tb_0.27_)_1−*y*_Fe*_y_* before and after heat treatment ((**a**) *y* = 0, (**b**) *y* = 0.05, (**c**) *y* = 0.10, (**d**) *y* = 0.15).

X-ray diffraction (XRD) experiments were performed for (Gd_0.73_Tb_0.27_)_1−*y*_Fe*_y_* alloys after heat treatment, as shown in Figure 7; the results showed that there was no new phase formed in (Gd_0.73_Tb_0.27_)_1−*y*_Fe*_y_* alloys compared with that before heat treatment. It means annealing at 1073 K for 10 h does not change the phase structure of the alloy, but only homogenizes the structure. Therefore, the improvement of magnetocaloric properties was due to the homogeneous composition of the sample after heat treatment, and the equilibrium phase was obtained, which is beneficial to the interaction among atomic magnetic moments.

## 4. Conclusions

In this work, the magnetocaloric properties of Gd_1*−x*_Tb*_x_* and (Gd_0.73_Tb_0.27_)_1*−y*_Fe*_y_* alloys were systematically studied.

- In Gd_1*−x*_Tb*_x_*, the Curie temperature decreased monotonously and linearly with the increase of Tb content, but the adiabatic temperature first rose and then decreased. Considering the magnetocaloric properties and the Curie temperature, *x* = 0.27 was the most suitable choice.
- In (Gd_0.73_Tb_0.27_)_1*−y*_Fe*_y_*, Fe doping reduced the adiabatic temperature change of the alloy while increasing the Curie temperature
- Heat treatment of (Gd_0.73_Tb_0.27_)_1*−y*_Fe*_y_* at 1073 K for 10 h resulted in an average increase in adiabatic temperature change of 0.3 K and a slight increase in the Curie temperature.
- The adiabatic temperature change obtained by the direct measurement method is widely used in the characterization of magnetocaloric effects. The results obtained by the direct measurement method had a good correlation with the results of the isothermal magnetic entropy change and the indirect measurement method, which just shows the accuracy of the direct measurement method.

The alloys studied in this paper have a high magnetocaloric effect, and doping Fe can effectively reduce the cost and adjust the Curie temperature. This series of alloys are potential magnetic refrigeration materials.
